# Pott's Puffy Tumor Presenting as Pyogenic Meningitis in an Infant

**DOI:** 10.1155/2022/4732287

**Published:** 2022-03-25

**Authors:** M. M. A. Faridi, Somya Pandey, Sumaiya Shamsi

**Affiliations:** ^1^Faculty of Medicine, Department of Pediatrics, Era's Lucknow Medical College & Hospital, Lucknow-226003, India; ^2^Department of Pediatrics, Era's Lucknow Medical College & Hospital, Lucknow-226003, India

## Abstract

*Introduction*. Pott's puffy tumor is characterized by the osteomyelitis of the frontal bone with underlying subperiosteal abscess, mostly occurring secondary to recurrent sinusitis or head trauma. Though it is a rare clinical entity in this antibiotic era, its occurrence mostly in the adolescent age group has now shown increased reporting lately in all age groups. *Case Description*. We describe here a case of a 4½-month-old female baby who presented to our hospital's Emergency Room with clinical features of pyogenic meningitis following aspiration of a midline frontal swelling. The infant presented with high-grade fever, 3-4 episodes of projectile vomiting, increased irritability, and refusal to breastfeeding than usual. This was accompanied by a history of a gradually increasing midline fluctuant erythematous swelling on her forehead extending to the left eye. Aspiration of the swelling was done a day before by a local general practitioner, following which she developed the above-mentioned features of pyogenic meningitis and was brought to the hospital the next day. Examination revealed a conscious, febrile, irritable child with bulging anterior fontanel and 101.4°F axillary temperature. Vital signs were within normal limits. CSF analysis was suggestive of pyogenic meningitis, and appropriate antibiotics were given. MRI showed frontal bone osteomyelitis with erosion of the bony plate and focal cerebritis. The condition turned out to be Pott's puffy tumor with pyogenic meningitis after detailed investigations. The infant was treated with appropriate antibiotics and other supportive therapeutic measures and discharged with the advice for further management in collaboration with otorhinolaryngologist.

## 1. Introduction

Sir Percivall Pott first described a rare clinical entity where the patient had frontal bone osteomyelitis with an associated subperiosteal abscess which might spread to the intracranial region either directly or through a hematogenous route. It was later on named as Pott's puffy tumor [1, 2]. The most common presenting symptoms of Pott's puffy tumor (PPT) include headache, fever, vomiting, and periorbital forehead swelling with tenderness (hence the term “puffy”). The patient may also complain of purulent nasal discharge with a history of sinusitis. Patients sometimes present with serious complications like pyogenic meningitis or intracranial abscess [3, 4]. The diagnosis is generally confirmed by computerized tomography of the skull.

Cases of PPT have been seen in all age groups; however, it occurs predominantly in adolescents and teenagers, which is believed to be due to peak vascularity in the diplopic circulation during adolescence [4, 5]. Recently, we came across an infant who had Pott's puffy tumor and presented with pyogenic meningitis. Our patient was only 4½ months old. This makes it a rare occurrence, perhaps the first case of its kind, in the literature. We are presenting the case with a brief review of the literature.

## 2. Case Description

A 4½-month-old female infant was brought to the Emergency Room in the night of the first week of October 2019 with a history of excessive crying, refusal to feed, fever, vomiting, and bulging anterior fontanelle.

The mother revealed that the child had a small midline swelling over the frontal bone near the left eyebrow since the age of 1½ months without pain or fever, which kept on growing for 15 days until one day she noticed purulent discharge from the left eye and left nostril; pus discharge continued for 15 days after which the swelling gradually reduced on its own. The infant was under treatment of a local doctor who prescribed some antibiotic eye drops, and the condition got relieved for some time. During this episode, the infant was afebrile and continued breastfeeding, though was uncomfortable.

**Figure 1:**
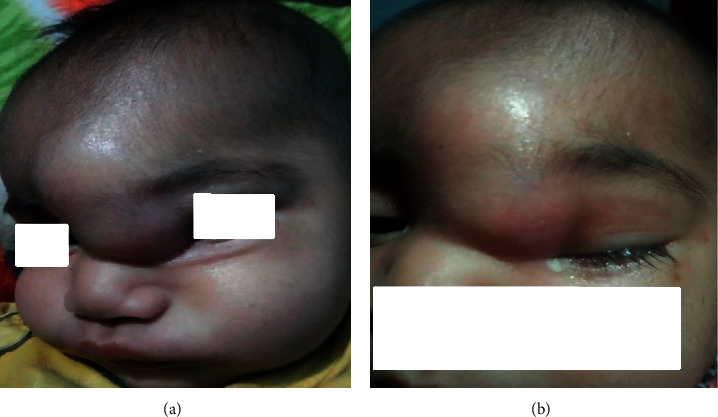
(a) Well-circumscribed swelling around the frontal bone area approximately (7 × 5 cm) involving left epicanthal fold and swelling of the left eyelid, which developed initially and subsided. The image obtained from the mother. (b) Swelling recurred after 4 weeks showing signs of inflammation. Purulent discharge from the left eye. The image obtained from the mother.

The swelling reappeared again at the same place about 2 weeks later. The infant was now febrile and very irritable. According to the mother, the swelling was of a peanut size that gradually progressed to the size of a tennis ball with redness and inflammation of the overlying skin. The description of the swelling matched with the photo taken by her on her mobile phone (Figures 1(a) and 1(b)). On further inquiry, she described swelling as fluctuant and boggy with well-defined margins. It was painful, but she could not recollect whether it was warm to touch. Parents consulted an ENT surgeon who did needle aspiration of the swelling, and the content was sent for Gram's staining and culture and sensitivity. The aspiration site was covered with small dressing gauze; oral antibiotics were prescribed, and the child was sent home.

On the same evening, the child developed high-grade fever, documented to 101°F with increased irritability, refusal to feed, and excessive crying and vomiting. The infant was brought to the Emergency Room of the hospital 6 hours after she threw a tonic and clonic convulsion. Injection phenytoin was administered at a dose of 20 mg/kg followed by maintenance at the dose of 5 mg/kg.

The child was conscious but was very irritable in the mother's lap. The anterior fontanelle was bulging and pulsatile, and the neck of the baby was retracted with increased tone in all four limbs. Physical examination on the admission day showed a well-nourished child, with heart rate 140/min, respiratory rate 38/min, axillary temperature 102.4°F, and blood pressure 100/70 mmHg (>95^th^ centile). The infant's weight was 5.2 kg (between median to -1SD), and length was 62 cm (50^th^ centile). Her head circumference was 39 cm (3^rd^ centile), pupils were bilaterally reactive and normal in shape and size, and there was no papilledema on fundoscopic examination. Facial strength and sensations were intact with no deviation of the mouth. The nasal passage was patent with healthy mucosa. There was no ear discharge. Pallor, cyanosis, maculopapular rash, icterus, or significant lymphadenopathies were not there. The aspiration site of the swelling (done outside) was found covered with a bandage near the glabella region. The area underneath the gauze was erythematous with small swelling around 1 × 1 cm near the left eyebrow which was tender to touch.

Central nervous examination revealed a conscious infant with intact cranial nerves. The motor system was within normal limits. The child had second seizure while examination was being done. Rest of the systemic examination did not reveal any abnormality. A provisional diagnosis of pyogenic meningitis with frontal sinus abscess was made.

Laboratory evaluation on the day of admission revealed random blood sugar 134 mg/dl, serum calcium 9.1 mg/dl, hemoglobin 11 mg/dl, polymorphonuclear leukocytosis (TLC 25,000; polymorphs 74%), platelet count 311,000/mm^3^, and erythrocyte sedimentation rate 22 mm in the first hour. The peripheral blood film was unremarkable; C-reactive protein was significantly elevated to 54 mg/L, while serum electrolytes were within normal limits (Na^+^ 136 mEq/L, K^+^ 3.6 mEq/L). Blood and urine culture sent on two different occasions were reported sterile. Cerebrospinal fluid analysis showed 1200 cells/mm^3^ (all polymorphs), 142 mg/dL proteins, and 30 mg/dL sugar against the blood sugar of 101 mg/dL. CSF culture was sterile for *Mycobacterium tuberculosis* and other organisms.

**Figure 2:**
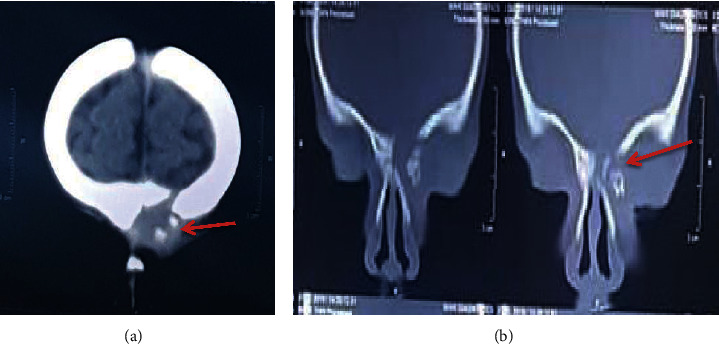
CT scan head of the infant. (a) Defect in the frontal bone; there is discontinuity of the left frontal bone with erosions. (b) Erosion of the frontal bone plate due to chronic postinfective etiology as seen on CT head, suggestive of PPT.

**Figure 3:**
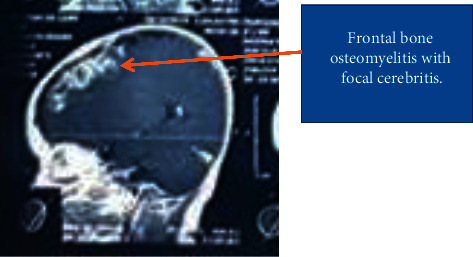
MRI brain of the infant in which left frontal bone osteomyelitis with focal cerebritis can be seen.

The child was referred by the ENT surgeon to the Emergency Room with a plain CT head done on the same day that showed postinfective osteomyelitis changes with erosion of the left frontal bone (Figure 2). The MRI brain and orbit were suggestive of chronic left frontal sinus osteomyelitis with focal cerebritis (Figure 3). There was no evidence of intracranial abscess.

### 2.1. Course of Hospital Stay

The child was admitted in the PICU, and orogastric emptying was done to prevent aspiration in case the infant throws a seizure again and kept nil orally. After obtaining blood culture, intravenous ceftriaxone (100 mg/kg/day × 12 hourly) and vancomycin (15 mg/kg/dose × three times a day) were started to cover *Haemophilus influenzae* and *Staphylococcus aureus* organisms. Intravenous N/2 dextrose saline was started as the daily maintenance fluid. Mannitol was given initially to reduce raised intracranial tension, and anticonvulsant was continued. Regular temperature, heart rate, respiratory rate, oxygen saturation, and blood pressure monitoring was done. The infant developed a high-grade fever of 102°F 8 hours after admission for which syrup paracetamol was given. The head circumference was measured daily to look for any evidence of hydrocephalus, and it was same at discharge.

Clinical improvement was seen from day 3 in the form of decrease in irritability, defervescence of fever, and acceptance of breastfeeding. There was reduction in the anterior fontanel bulge. IV fluids were tapered and stopped in the next 24 hours.

**Figure 4:**
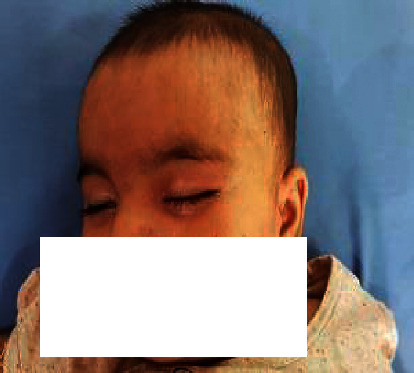
On discharge, the infant clinically improved.

On the 4^th^ day of admission, the child was active, playful, afebrile, and accepting breastfeeds well; the anterior fontanel was at level (Figure 4). The patient responded remarkably to the therapy and antibiotics, and antiepileptics were stopped after 14 days. There was no recurrence of the swelling.

The child was discharged after 15 days on oral antibiotics (linezolid and augmentin for 2 weeks) with the advice for follow-up after one week, along with need for surgical consideration for the underlying frontal bone erosion. However, the patient was lost to follow-up till recently the mother shared some photos of the child by Whatsapp. The child was doing fine. On enquiry, the mother did not share the reason for loss to follow-up.

## 3. Discussion

Pott's puffy tumor (PPT), as described by Sir Percivall Pott is a “puffy,” circumscribed, indolent tumor of the scalp with spontaneous separation of the pericranium from the skull [3]. Specifically, it is frontal sinus osteomyelitis with associated subperiosteal abscess of the frontal bone, with acute or subacute presentation. The anterior table of the frontal sinus is thinner than the posterior table and is more susceptible to abscess formation. When the posterior table of the frontal sinus is eroded, then infection tends to spread to the brain and may cause epidural/subdural empyema, meningitis, cavernous/sagittal sinus thrombosis, and intracranial abscess. Locally, it can lead to orbital complications like cellulitis or abscess [4, 5].

PPT initially described with head trauma is now known to be associated with untreated or partially treated sinusitis or rarely due to mastoid surgery, dental infection, and sometimes insect bite [6]. Both Pott's spine and Pott's puffy tumor have been described by Sir Percivall Pott, a British surgeon, but Pott's puffy tumor has no clinical relation with Pott's spine which is an extrapulmonary TB involvement of the spine.

In our case, PPT presented as pyogenic meningitis, although the child might have been suffering from chronic frontal sinus osteomyelitis which led to abscess formation and spontaneous rupture with pus drainage through the left nostril and left eye. The imaging modalities revealed erosion and destruction of the frontal bone, as a result of chronic osteomyelitis, and focal cerebritis of the left frontal lobe.

To the best of our knowledge and search of the literature, we can say that this is a very early presentation of Pott's puffy tumor in an infant. In pediatric population, PPT usually presents in children and teenagers with pneumatization of the frontal sinuses. Age demographics are reported at a wide range from the first through eighth decades of life, though teenagers and adolescents are predominately affected [7, 8]. Most cases present as frontal headache, vomiting, and midline frontal swelling.

The youngest patient of PPT reported in the literature was a 5-year-old girl, who had frontal headache, decreased appetite, and fever for a month with progressive frontal swelling, diagnosed as PPT on CT head, and was initially treated with a 12-day course of intravenous antibiotics. The neurological examination was normal with full painless extraocular movements. CT findings were consistent with orbital cellulitis with underlying osteomyelitis and invasion of the superior sagittal sinus. Investigations showed mild leukocytosis (12.5 × 10^9^ cells/L) and raised CRP (133 mg/L). Finally, she was operated with drainage of abscess and recovered fully with a 12-week course of intravenous antibiotics after surgery [9].

Similar cases of PPT have been reported in different age groups; an 8-year-old girl presenting with recurrent URTI and forehead swelling with persistent headache was initially treated with azithromycin. CT findings revealed frontal sinusitis with cortical erosions and had to undergo surgery later on [10].

A 10-year-old girl presented with seizures following subperiosteal abscess of frontal bone [11]. A 13-year-old boy presented with significant seasonal allergy, prolonged headache, and concomitant forehead swelling, reported in June 2019 [12]. Most of them had intracranial complications and recovered with intravenous antibiotics, while in some cases, surgical drainage and removal of the destructive osteolytic bone with functional endoscopic sinus surgery (FESS) were required.

Cases have been reported in older age groups as well. A 70-year-old man with a history of prolonged nasal obstruction, hyposmia, and recurrent sinusitis with no previous treatment developed a reddish tender bulge on the forehead after sustaining an injury to his head 6 weeks ago. CT findings revealed anterior and posterior frontal sinus table bone destruction communicating intracranially and subperiosteally which was later confirmed by MRI. WBC and CRP were not elevated. Surgery was done under antibiotic coverage with cefuroxime and metronidazole. He remained symptom free for 1 year after surgery on follow-up.

## 4. Conclusion

PPT can be present in different age groups in various forms. In our case, presenting symptoms were suggestive of pyogenic meningitis, although the underlying cause was PPT, which responded well to antibiotics. CT scan brain is a diagnostic modality of choice. In the present case, there was intracranial and extracranial fluid collection, with erosion of the frontal bone with underlying chronic postinfective etiology, which is consistent with PPT. Presently, broad-spectrum antibiotics along with surgical drainage is the standard of care.

It is sometimes a surgical emergency, and with the advent of functional endoscopic sinus surgery (FESS), this treatment can often be performed with minimal invasion [13]. Ideally, the destructed bone and granulation tissue require removal which is better through FESS. Milder cases can be treated with a 72-hour trial of intranasal corticosteroid. However, in our case, it responded well to antibiotics only.

### 4.1. “Take-Away” Lesson

Pediatrician and family physician should thoroughly examine a child presenting with purulent eye discharge, sinusitis, or swelling over the forehead lest one misses PPT, a potentially serious complication of chronic sinusitis.

## Data Availability

The data on Pott's puffy tumor used to support the findings of this study are included within the article.

## Additional Points

The case is presented with a brief review of the literature as it is uncommon in infancy; family physician and pediatricians should examine thoroughly any child presenting with frontal midline swelling keeping high suspicion for Pott's puffy tumor. This case highlights the need to recognize and prevent fatal complication of a seemingly benign condition.
